# *N*-Carbamothioyl­amino-7-oxabicyclo­[2.2.1]hept-5-ene-2,3-dicarboximide

**DOI:** 10.1107/S160053681004835X

**Published:** 2010-11-27

**Authors:** Jian Li

**Affiliations:** a Department of Chemistry and Chemical Engineering, Weifang University, Weifang 261061, People’s Republic of China

## Abstract

The title compound, C_9_H_9_N_3_O_3_S, comprises a racemic mixture of chiral mol­ecules containing four stereogenic centres. The cyclo­hexane ring tends towards a boat conformation, while the tetra­hydro­furan ring and the dihydro­furan ring adopt envelope conformations. The dihedral angle between the thio­semicarbazide fragment and the fused-ring system is 77.20 (10)°. The crystal structure is stabilized by two inter­molecular N—H⋯O hydrogen bonds.

## Related literature

For the use of 7-oxa-bicyclo­[2,2,1]hept-5-ene-2,3-dicarb­oxy­lic anhydride in clinical practice, see: Deng & Hu (2007 ▶). For the pharmacological activity of its derivatives, see: Hart *et al.* (2004 ▶). For bond lengths and angles in related structures, see: Goh *et al.* (2008 ▶).
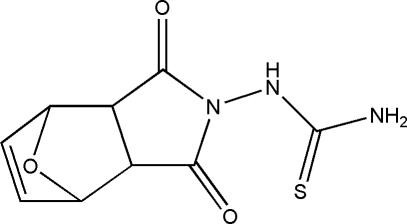


## Experimental

### 

#### Crystal data

C_9_H_9_N_3_O_3_S
*M* *_r_* = 239.25
Orthorhombic, 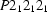

*a* = 8.3978 (8) Å
*b* = 8.9032 (9) Å
*c* = 13.5930 (14) Å
*V* = 1016.31 (18) Å^3^
*Z* = 4
Mo *K*α radiation
μ = 0.31 mm^−1^
*T* = 298 K
0.45 × 0.43 × 0.40 mm

#### Data collection

Bruker SMART CCD area-detector diffractometer
Absorption correction: multi-scan (*SADABS*; Bruker, 1997 ▶) *T*_min_ = 0.872, *T*_max_ = 0.885
5015 measured reflections
1791 independent reflections
1632 reflections with *I* > 2σ(*I*)
*R*_int_ = 0.023

#### Refinement

*R*[*F* ^2^ > 2σ(*F* ^2^)] = 0.029
*wR*(*F* ^2^) = 0.071
*S* = 1.07
1791 reflections
145 parameters
H-atom parameters constrained
Δρ_max_ = 0.14 e Å^−3^
Δρ_min_ = −0.16 e Å^−3^
Absolute structure: Flack (1983 ▶), 728 Friedel pairs
Flack parameter: 0.01 (9)

### 

Data collection: *SMART* (Bruker, 1997 ▶); cell refinement: *SAINT* (Bruker, 1997 ▶); data reduction: *SAINT*; program(s) used to solve structure: *SHELXS97* (Sheldrick, 2008 ▶); program(s) used to refine structure: *SHELXL97* (Sheldrick, 2008 ▶); molecular graphics: *SHELXTL* (Sheldrick, 2008 ▶); software used to prepare material for publication: *SHELXTL*.

## Supplementary Material

Crystal structure: contains datablocks global, I. DOI: 10.1107/S160053681004835X/bx2331sup1.cif

Structure factors: contains datablocks I. DOI: 10.1107/S160053681004835X/bx2331Isup2.hkl

Additional supplementary materials:  crystallographic information; 3D view; checkCIF report

## Figures and Tables

**Table 1:** Hydrogen-bond geometry (Å, °)

*D*—H⋯*A*	*D*—H	H⋯*A*	*D*⋯*A*	*D*—H⋯*A*
N2—H2⋯O3^i^	0.86	1.96	2.809 (2)	167
N3—H3*B*⋯O1^ii^	0.86	2.14	2.958 (2)	160

Symmetry codes: (i) 
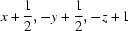
; (ii) 
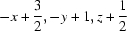
.

**Fig. 1.**
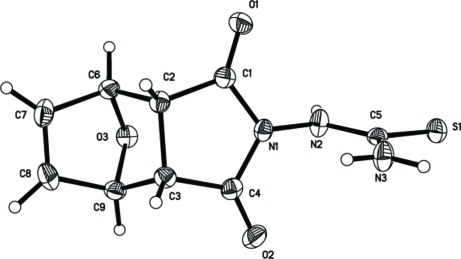
The molecular structure of (I), with the atom-numbering scheme. Displacement ellipsoide are drawn at 30% probability level.

**Fig. 2.**
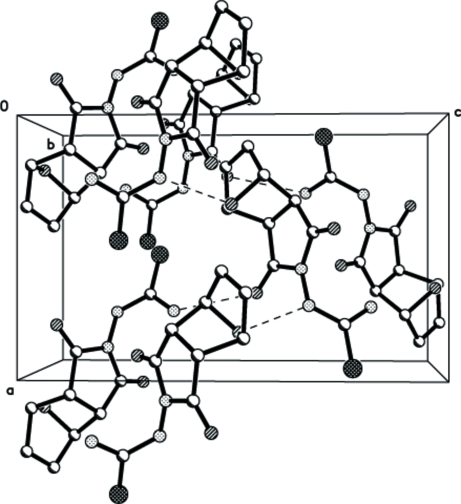
The crystal packing of (I), viewed along b axis. Dashed lines indicate hydrogen bonds.

## supplementary crystallographic information

### Comment

7-Oxa-bicyclo[2,2,1]hept-5-ene-2,3-dicarboxylic anhydride has been widely employed in clinical practice, as it is less toxic and much easier to be synthesized [Deng *et al.*, 2007]. Its derivatives are also pharmacologically active [Hart *et al.*, 2004]. We report here the crystal structure of the title compound, (I) which comprises a racemic mixture of chiral molecules containing four stereogenic centres. The cyclohexane ring tends towards a boat conformation, the tetrahydrofuran ring and the dihydrofuran ring adopt envelope conformations (Fig. 1). The bond lengths and bond angles are normal range and comparable to those in the similar compound [Goh, *et al.*, 2008] as representative example. The dihedral angle between the thiosemicarbazide fragment and fused-ring system is 77.20 (10)°. The crystal structure is stabilized by two intermolecular N—H···O and one intramolecular N—H···N hydrogen bonds (Table 1, Fig. 2).

### Experimental

A mixture of *exo*-7-oxa-bicyclo[2,2,1]hept-5-ene-2,3-dicarboxylic anhydride (0.332 g, 2 mmol) and thiocarbanide (0.182 g, 2 mmol) in methanol (5 ml) was stirred for 5 h at room temperature, and then refluxed for 1 h. After cooling the precipitate was filtered and dried, the title compound was obtained. The crude product of 20 mg was dissolved in methanol of 10 ml. The solution was filtered to remove impurities, and then the filtrate was left for crystallization at room temperature. The single-crystal suitable for X-ray determination was obtained by evaporation from the methanol solution after 5 d.

### Refinement

H atoms were initially located from difference maps and then refined in a riding model with C—H = 0.93–0.96 Å and *U*_iso_(H) = 1.2*U*_eq_(C) or 1.5*U*_eq_(methyl C).

### Crystal data

**Table:**

C_9_H_9_N_3_O_3_S	*F*(000) = 496
*M**_r_* = 239.25	*D*_x_ = 1.564 Mg m^−^^3^
Orthorhombic, *P*2_1_2_1_2_1_	Mo *K*α radiation, λ = 0.71073 Å
Hall symbol: P 2ac 2ab	Cell parameters from 2624 reflections
*a* = 8.3978 (8) Å	θ = 2.7–26.3°
*b* = 8.9032 (9) Å	µ = 0.31 mm^−^^1^
*c* = 13.5930 (14) Å	*T* = 298 K
*V* = 1016.31 (18) Å^3^	Block, light yellow
*Z* = 4	0.45 × 0.43 × 0.40 mm

### Data collection

**Table:**

Bruker SMART CCD area-detector diffractometer	1791 independent reflections
Radiation source: fine-focus sealed tube	1632 reflections with *I* > 2σ(*I*)
graphite	*R*_int_ = 0.023
phi and ω scans	θ_max_ = 25.0°, θ_min_ = 2.7°
Absorption correction: multi-scan (*SADABS*; Bruker, 1997)	*h* = −9→9
*T*_min_ = 0.872, *T*_max_ = 0.885	*k* = −10→10
5015 measured reflections	*l* = −16→11

### Refinement

**Table:**

Refinement on *F*^2^	Secondary atom site location: difference Fourier map
Least-squares matrix: full	Hydrogen site location: inferred from neighbouring sites
*R*[*F*^2^ > 2σ(*F*^2^)] = 0.029	H-atom parameters constrained
*wR*(*F*^2^) = 0.071	*w* = 1/[σ^2^(*F*_o_^2^) + (0.0346*P*)^2^ + 0.1693*P*] where *P* = (*F*_o_^2^ + 2*F*_c_^2^)/3
*S* = 1.07	(Δ/σ)_max_ < 0.001
1791 reflections	Δρ_max_ = 0.14 e Å^−^^3^
145 parameters	Δρ_min_ = −0.16 e Å^−^^3^
0 restraints	Absolute structure: Flack (1983), 728 Friedel pairs
Primary atom site location: structure-invariant direct methods	Flack parameter: 0.01 (9)

### Special details

**Table:**

Geometry. All e.s.d.'s (except the e.s.d. in the dihedral angle between two l.s. planes) are estimated using the full covariance matrix. The cell e.s.d.'s are taken into account individually in the estimation of e.s.d.'s in distances, angles and torsion angles; correlations between e.s.d.'s in cell parameters are only used when they are defined by crystal symmetry. An approximate (isotropic) treatment of cell e.s.d.'s is used for estimating e.s.d.'s involving l.s. planes.
Refinement. Refinement of *F*^2^ against ALL reflections. The weighted *R*-factor *wR* and goodness of fit *S* are based on *F*^2^, conventional *R*-factors *R* are based on *F*, with *F* set to zero for negative *F*^2^. The threshold expression of *F*^2^ > σ(*F*^2^) is used only for calculating *R*-factors(gt) *etc*. and is not relevant to the choice of reflections for refinement. *R*-factors based on *F*^2^ are statistically about twice as large as those based on *F*, and *R*- factors based on ALL data will be even larger.

### Fractional atomic coordinates and isotropic or equivalent isotropic displacement parameters (Å^2^)

**Table:**

	*x*	*y*	*z*	*U*_iso_*/*U*_eq_	
S1	0.97059 (7)	0.24096 (7)	0.78384 (4)	0.04347 (17)	
N1	0.5849 (2)	0.39754 (19)	0.65892 (11)	0.0314 (4)	
N2	0.7287 (2)	0.3264 (2)	0.67804 (13)	0.0407 (5)	
H2	0.7712	0.2722	0.6327	0.049*	
N3	0.7384 (2)	0.4298 (2)	0.83195 (13)	0.0447 (5)	
H3A	0.6516	0.4766	0.8184	0.054*	
H3B	0.7826	0.4413	0.8885	0.054*	
O1	0.68615 (19)	0.5596 (2)	0.54443 (11)	0.0466 (4)	
O2	0.4254 (2)	0.22814 (19)	0.73858 (12)	0.0529 (5)	
O3	0.32651 (18)	0.33700 (17)	0.48808 (10)	0.0362 (4)	
C1	0.5731 (3)	0.5099 (2)	0.58861 (14)	0.0321 (5)	
C2	0.4001 (2)	0.5466 (2)	0.57606 (14)	0.0306 (5)	
H2A	0.3754	0.6517	0.5911	0.037*	
C3	0.3110 (2)	0.4351 (2)	0.64312 (15)	0.0322 (5)	
H3	0.2452	0.4855	0.6927	0.039*	
C4	0.4385 (3)	0.3383 (2)	0.68800 (14)	0.0334 (5)	
C5	0.8043 (3)	0.3395 (2)	0.76542 (14)	0.0298 (5)	
C6	0.3396 (3)	0.4970 (3)	0.47264 (15)	0.0357 (5)	
H6	0.4065	0.5276	0.4171	0.043*	
C7	0.1677 (3)	0.5434 (3)	0.46640 (18)	0.0457 (6)	
H7	0.1252	0.6207	0.4287	0.055*	
C8	0.0896 (3)	0.4521 (3)	0.52523 (17)	0.0450 (6)	
H8	−0.0191	0.4531	0.5382	0.054*	
C9	0.2106 (3)	0.3459 (3)	0.56743 (15)	0.0368 (5)	
H9	0.1687	0.2495	0.5906	0.044*	

### Atomic displacement parameters (Å^2^)

**Table:**

	*U*^11^	*U*^22^	*U*^33^	*U*^12^	*U*^13^	*U*^23^
S1	0.0357 (3)	0.0469 (3)	0.0479 (3)	0.0058 (3)	−0.0064 (3)	0.0046 (3)
N1	0.0315 (9)	0.0367 (10)	0.0261 (8)	0.0045 (8)	−0.0032 (8)	−0.0030 (8)
N2	0.0382 (10)	0.0540 (12)	0.0299 (10)	0.0176 (9)	−0.0048 (8)	−0.0105 (8)
N3	0.0447 (11)	0.0553 (12)	0.0342 (10)	0.0059 (10)	−0.0116 (9)	−0.0121 (9)
O1	0.0357 (9)	0.0642 (12)	0.0399 (9)	−0.0093 (8)	0.0035 (8)	0.0077 (8)
O2	0.0607 (10)	0.0508 (10)	0.0472 (9)	−0.0104 (9)	−0.0100 (8)	0.0191 (9)
O3	0.0405 (8)	0.0351 (8)	0.0330 (8)	0.0003 (7)	−0.0017 (7)	−0.0079 (7)
C1	0.0368 (12)	0.0350 (11)	0.0245 (10)	−0.0046 (10)	−0.0007 (9)	−0.0048 (9)
C2	0.0333 (11)	0.0257 (11)	0.0329 (11)	0.0010 (9)	−0.0030 (10)	−0.0008 (9)
C3	0.0324 (11)	0.0354 (12)	0.0287 (10)	0.0009 (9)	0.0042 (9)	−0.0030 (9)
C4	0.0411 (13)	0.0348 (12)	0.0243 (10)	−0.0023 (10)	−0.0010 (9)	−0.0046 (9)
C5	0.0330 (11)	0.0283 (10)	0.0282 (11)	−0.0068 (9)	0.0014 (9)	0.0015 (9)
C6	0.0360 (11)	0.0411 (13)	0.0300 (11)	−0.0061 (11)	−0.0019 (9)	0.0050 (10)
C7	0.0449 (14)	0.0464 (15)	0.0459 (13)	0.0038 (12)	−0.0185 (12)	0.0023 (11)
C8	0.0292 (11)	0.0590 (17)	0.0469 (13)	−0.0001 (11)	−0.0064 (11)	−0.0078 (12)
C9	0.0352 (12)	0.0405 (12)	0.0345 (12)	−0.0095 (10)	0.0001 (10)	0.0027 (10)

### Geometric parameters (Å, °)

**Table:**

S1—C5	1.668 (2)	C2—C3	1.542 (3)
N1—C1	1.387 (3)	C2—C6	1.558 (3)
N1—N2	1.388 (2)	C2—H2A	0.9800
N1—C4	1.395 (3)	C3—C4	1.504 (3)
N2—C5	1.352 (3)	C3—C9	1.549 (3)
N2—H2	0.8600	C3—H3	0.9800
N3—C5	1.331 (3)	C6—C7	1.505 (3)
N3—H3A	0.8600	C6—H6	0.9800
N3—H3B	0.8600	C7—C8	1.315 (3)
O1—C1	1.207 (2)	C7—H7	0.9300
O2—C4	1.203 (2)	C8—C9	1.502 (3)
O3—C6	1.444 (3)	C8—H8	0.9300
O3—C9	1.455 (3)	C9—H9	0.9800
C1—C2	1.499 (3)		
			
C1—N1—N2	121.34 (18)	O2—C4—N1	123.4 (2)
C1—N1—C4	113.89 (17)	O2—C4—C3	129.3 (2)
N2—N1—C4	122.77 (17)	N1—C4—C3	107.21 (16)
C5—N2—N1	122.28 (18)	N3—C5—N2	116.96 (19)
C5—N2—H2	118.9	N3—C5—S1	124.34 (16)
N1—N2—H2	118.9	N2—C5—S1	118.69 (16)
C5—N3—H3A	120.0	O3—C6—C7	101.85 (18)
C5—N3—H3B	120.0	O3—C6—C2	99.98 (16)
H3A—N3—H3B	120.0	C7—C6—C2	106.61 (17)
C6—O3—C9	96.03 (15)	O3—C6—H6	115.5
O1—C1—N1	123.4 (2)	C7—C6—H6	115.5
O1—C1—C2	128.8 (2)	C2—C6—H6	115.5
N1—C1—C2	107.75 (17)	C8—C7—C6	105.9 (2)
C1—C2—C3	105.23 (17)	C8—C7—H7	127.0
C1—C2—C6	110.89 (17)	C6—C7—H7	127.0
C3—C2—C6	101.12 (16)	C7—C8—C9	106.5 (2)
C1—C2—H2A	112.9	C7—C8—H8	126.7
C3—C2—H2A	112.9	C9—C8—H8	126.7
C6—C2—H2A	112.9	O3—C9—C8	101.75 (17)
C4—C3—C2	105.28 (17)	O3—C9—C3	99.01 (15)
C4—C3—C9	111.29 (18)	C8—C9—C3	107.42 (18)
C2—C3—C9	101.62 (16)	O3—C9—H9	115.5
C4—C3—H3	112.6	C8—C9—H9	115.5
C2—C3—H3	112.6	C3—C9—H9	115.5
C9—C3—H3	112.6		
			
C1—N1—N2—C5	114.5 (2)	C9—C3—C4—N1	115.12 (18)
C4—N1—N2—C5	−82.6 (3)	N1—N2—C5—N3	−3.0 (3)
N2—N1—C1—O1	−4.8 (3)	N1—N2—C5—S1	176.11 (16)
C4—N1—C1—O1	−169.10 (19)	C9—O3—C6—C7	−49.14 (18)
N2—N1—C1—C2	171.97 (16)	C9—O3—C6—C2	60.34 (17)
C4—N1—C1—C2	7.6 (2)	C1—C2—C6—O3	76.2 (2)
O1—C1—C2—C3	173.2 (2)	C3—C2—C6—O3	−34.98 (19)
N1—C1—C2—C3	−3.4 (2)	C1—C2—C6—C7	−178.11 (19)
O1—C1—C2—C6	64.6 (3)	C3—C2—C6—C7	70.7 (2)
N1—C1—C2—C6	−111.89 (18)	O3—C6—C7—C8	32.6 (2)
C1—C2—C3—C4	−1.5 (2)	C2—C6—C7—C8	−71.7 (2)
C6—C2—C3—C4	113.97 (17)	C6—C7—C8—C9	−1.1 (2)
C1—C2—C3—C9	−117.64 (18)	C6—O3—C9—C8	48.43 (18)
C6—C2—C3—C9	−2.2 (2)	C6—O3—C9—C3	−61.60 (17)
C1—N1—C4—O2	170.38 (19)	C7—C8—C9—O3	−30.6 (2)
N2—N1—C4—O2	6.3 (3)	C7—C8—C9—C3	72.9 (2)
C1—N1—C4—C3	−8.6 (2)	C4—C3—C9—O3	−73.4 (2)
N2—N1—C4—C3	−172.69 (17)	C2—C3—C9—O3	38.29 (19)
C2—C3—C4—O2	−173.1 (2)	C4—C3—C9—C8	−178.77 (18)
C9—C3—C4—O2	−63.8 (3)	C2—C3—C9—C8	−67.1 (2)
C2—C3—C4—N1	5.8 (2)		

### Hydrogen-bond geometry (Å, °)

**Table:**

*D*—H···*A*	*D*—H	H···*A*	*D*···*A*	*D*—H···*A*
N2—H2···O3^i^	0.86	1.96	2.809 (2)	167
N3—H3B···O1^ii^	0.86	2.14	2.958 (2)	160
N3—H3A···N1	0.86	2.35	2.697 (2)	105

Symmetry codes: (i) *x*+1/2, −*y*+1/2, −*z*+1; (ii) −*x*+3/2, −*y*+1, *z*+1/2.
